# Evaluation of a maternal health care project in South West Shoa Zone, Ethiopia: before-and-after comparison

**DOI:** 10.1186/s12978-016-0213-1

**Published:** 2016-08-20

**Authors:** Calistus Wilunda, Shiro Tanaka, Giovanni Putoto, Ademe Tsegaye, Koji Kawakami

**Affiliations:** 1 Department of Pharmacoepidemiology, Graduate School of Medicine and Public Health, Kyoto University, Yoshida Konoecho Sakyoku, Kyoto, 606-8501 Japan; 2 Doctors with Africa CUAMM, Via San Francesco 126, 35121 Padua, Italy; 3 Doctors with Africa CUAMM, P.O Box 12777, Addis Ababa, Ethiopia

**Keywords:** Ethiopia, Maternal health, Project evaluation, Skilled birth attendance, Antenatal care

## Abstract

**Background:**

Despite recent achievements in health targets, Ethiopia still faces challenges in health service delivery. Between 2012 and 2015, a non-governmental organisation (NGO), Doctors with Africa CUAMM, implemented a multifaceted project aimed at improving access to maternal and child health services in three districts in Ethiopia. This paper evaluates the performance of this project, based on four maternal health indicators.

**Methods:**

A before-and-after study utilising data collected through cross-sectional surveys involving 999 women was conducted. The date of delivery was used to stratify the intervention period as follows: pre-intervention, early intervention, and late intervention. Changes during the intervention in the coverage of four antenatal care (ANC) visits, receipt of three basic components of ANC, skilled birth attendant (SBA) at delivery, and postnatal care (PNC) in seven days were assessed using logistic regression, adjusting for socio-demographic factors.

**Results:**

There was an increase in the coverage of receipt of all three ANC components and SBA at delivery between the pre-intervention period and the late intervention period. The percent of health centre deliveries increased from 7.3 % in the pre-intervention period to 35.6 % in the late intervention period. The odds of receiving all three components of ANC were twice higher in the late intervention period than in the pre-intervention period (OR 2.09; 95 % CI 1.12–3.89). The odds of SBA at delivery were five times higher in the late intervention period than in the pre-intervention period (OR 5.04; 95 % CI 2.53–10.06). There was no significant change in the coverage of four ANC visits and PNC after accounting for sociodemographic factors.

**Conclusions:**

This NGO implemented maternal health project in three districts in Ethiopia was associated with increased likelihood that a pregnant woman would receive three basic components of ANC and be assisted by a SBA at delivery. Increase in skilled birth attendance was driven by increased utilisation of health centres. More efforts are needed to bolster the coverage of ANC and PNC.

**Electronic supplementary material:**

The online version of this article (doi:10.1186/s12978-016-0213-1) contains supplementary material, which is available to authorized users.

## Background

Maternal mortality ratio (per 100,000 live births) is estimated to have significantly declined in Ethiopia, from 1,250 in 1990 to 353 in 2015 (a 72 % drop); just shy of achieving the Millennium Development Goal (MGD) 5 target of 75 % reduction [1]. Ethiopia has also made remarkable achievements in reducing child mortality; the country achieved its MDG 4 target of reducing child deaths by two thirds between 1990 and 2015 [2]. Despite these achievements, the number of maternal deaths in Ethiopia is still high; the country is one of the ten countries that contribute to 59 % of global maternal deaths [1]. Ethiopia also has a disproportionately high number of neonatal deaths; 43 % of the under-5 deaths are neonatal deaths [3].

The high maternal and neonatal mortality reflect poor coverage of maternal and neonatal health care services, poor quality of care provided in health facilities, and inequity in access to health services. Coverage of the recommended minimum four antenatal care (ANC) visits increased from 19 % in the 2011 Demographic and Health Survey (DHS) to 32 % in the 2014 DHS survey, and that of skilled birth attendant (SBA) at delivery correspondingly increased from 10 to 16 % [4, 5]. Ethiopia is one of the six countries where more than half of the mothers and children in the poorest 20 % of the population receive two or fewer of eight essential interventions for preventing maternal and child deaths [3]. The reasons behind the high maternal and neonatal mortality in Ethiopia have been explained using the “three delays” model [6].

Various health system constraints affect maternal health service delivery in Ethiopia. These include inadequate basic health infrastructure, shortage of skilled staff, weak referral systems, limited availability of equipment, limited financing for services, weak management, poor staff motivation, and weaknesses in implementation of government programs [7, 8]. The density of doctors, nurses and midwives per 10,000 population in the country was 6.3 in 2012/2013 [9]; way below the 23 recommended by WHO [10], and the per capita total expenditure on health was 44 US$ in 2012 [3]. A substantial amount of healthcare funding comes from donors; in 2011, Ethiopia received the second highest share (6.1 %) of the total official development assistance for maternal, neonatal and child health [3].

Doctors with Africa CUAMM (http://www.mediciconlafrica.org/), hereafter referred to as CUAMM, is an Italian non-governmental organisation (NGO) that has been supporting health service management and delivery in Ethiopia since 1984. CUAMM’s current strategy is based on the continuum of care approach [11]. Between 2012 and 2015, CUAMM implemented a multifaceted maternal and child health project in three districts (so called *woredas*) in South West Shoa Zone, Oromia region. The project aimed to improve access to maternal and child health services through tackling demand and supply side barriers to service access; focusing mainly on health centres (HCs) and the community. Key determinants of maternal health service access and utilisation in the districts include distance to health facilities, attitude towards maternal health care, knowledge of maternal health, perceived quality of maternal health services, involvement of the family members in decision making on delivery place, and birth preparedness [12]. Additionally, there is stack inequity in utilisation of maternal health services in the districts by wealth status and urban/rural residence [12, 13].

This study aimed to evaluate the effect of this project on access to essential maternal and neonatal healthcare services including ANC, delivery by a skilled provider and postnatal care (PNC).

## Methods

### Setting

The project was implemented in Wolisso, Goro and Wonchi districts of South West Shoa Zone, Oromia region in central Ethiopia. The districts are located about 115 km south-west of Addis Ababa, the capital of Ethiopia. The three districts had a combined population of about 398,000 inhabitants in 2014 and are served by one hospital (St. Luke Catholic Hospital), which also acts as a zonal referral hospital, 18 HCs and 89 health posts (HPs). The hospital is a private non-profit facility and hence had a system of user fees before the project began. In Ethiopia, maternity services are usually provided at hospitals and HCs. HCs, which are designed to serve a catchment population of 25,000 people, are expected to provide a full range of routine maternal health services plus emergency obstetric care services except blood transfusion and caesarean section, which can only be provided at hospital level [14]. HPs are run by salaried health extension workers (HEWs) who are mainly female community members with high school-level education and have been trained for one year to provide preventive, promotive and selective curative health services. HEWs increase the knowledge and skills of communities to deal with preventable diseases and to utilise health services provided at HCs and hospitals, and also provide care to women during pregnancy, childbirth and postnatal periods either in HPs or in households [14–16]. Thus, they spend about 75 % of their time conducting outreach activities and the rest at HPs. All the HCs and HPs in the study area are government owned and provide maternal health services free-of-charge as per the national policy.

### Description of the project

The project was embedded in the health system of the districts, and during its course, the following activities were conducted to improve maternal and neonatal health care:

1. The zonal health office received technical and material support including office construction and furnishing, strengthening of the health information system including support in data analysis and use for planning, and support in coordination of meetings and in monitoring of maternal and neonatal health care activities.
2. HCs were rehabilitated and the infrastructure was improved. This included equipping maternity wards with the missing medical equipment and providing generators/solar panels and running water to ensure 24-h availability of health services.
3. HCs received a regular supply of consumable supplies and drugs to supplement what was being received from the government.
4. Health workers were trained on maternal and neonatal health care including ANC, intrapartum care, PNC and emergency obstetrics and neonatal care. The trainings were conducted by staff from the Department of Obstetrics and Gynaecology, St. Luke Catholic Hospital.
5. Staff members of HCs were supervised supportively with the aim of identifying and addressing their work-related challenges. A standard checklist was developed to guide the supervision.
6. All health extension workers (150 in total) received refresher trainings according to national guidelines. The trainings were conducted at a central location by project staff in collaboration with staff from the Department of Paediatrics, St. Luke Catholic Hospital. HEWs were then supervised using a standard checklist by trained supervisors based at HCs.
7. The referral system was strengthened through provision of free-of-charge ambulance service, provision of communication equipment at HCs, and training of staff on referral protocols. The ambulance was based at the hospital and was used to transfer pregnant women from villages to HCs and, if required, from the HCs to the hospital. The ambulance could be accessed by calling either the phone number specifically designated for the ambulance, or the hospital. Details about the ambulance service and the referral system are available elsewhere [17].
8. All user fees including fees for management of obstetric and neonatal complications and caesarean section at the hospital were removed.
9. Community sensitization activities were conducted through strengthening village (*kebele*) command posts which comprise of HEWs and village level leaders. The aim was to increase demand for maternal, neonatal and child health services in the villages. Other sensitization activities included radio broadcasts about available free-of-charge services, and distribution of maternal health information, education and communication materials.

A detailed work plan guided the implementation of the project. Monitoring of the project was conducted jointly by CUAMM and local partners (zonal and district health authorities) through quarterly review meetings, quarterly activity and financial reports, planned field visits and supportive supervision.

### Design and study population

This study utilised before-and-after intervention design based on data collected through two cross-sectional surveys. The study population consisted of women of reproductive age who delivered within two years preceding each survey, in the study districts.

### Data collection

Data were collected through household surveys conducted in February 2013 and March 2015. The surveys utilised similar methods and tools (questionnaires). The questionnaires were adapted from the UNICEF’s Multiple Cluster Indicator Survey questionnaires and JHPIEGO’s tools for monitoring birth preparedness and complication readiness [18], and were pretested and translated into Oromo language. During each survey, women who delivered within two years preceding each survey were asked questions related to care during pregnancy, delivery and after delivery of the youngest child. Data were also collected on household and socio-demographic characteristics, birth preparedness, knowledge of pregnancy related danger signs, perceptions towards maternal health care and perceived quality of care. The surveys utilised multistage sampling using a modified Expanded Program for Immunisation’s random walk method [19] to select study subjects. The first stage involved selection of villages and the second stage involved selection of eligible women in the selected village. Details of the sampling method are available elsewhere [12].

### Sample size

The first survey collected data from a sample of 500 women estimated assuming institutional delivery coverage of 20 %, an absolute precision of 0.05, and a Z score value of 1.96 for 95 % confidence interval and a design effect of 2. Due to limited resources, the second survey included a similar number of women. This evaluation was sufficiently powered (>95 %) to detect significant differences at 5 % alpha level between the pre-intervention period and the late intervention period for all the outcomes except for PNC as shown in the Additional file 1.

### Definition of intervention periods

**Fig. 1:**
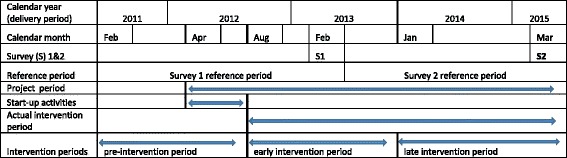
Timeline of the project and household surveys (not drawn to scale)

Each survey had a reference period of preceding two years (Fig. 1). This implies that the reference period of the surveys was the entire duration of the project plus a period of 14 months before the start. Although the project began in April 2012, the first four months were spent on preparatory activities such as hiring of staffs and procurement of supplies, and so the actual intervention period began in August 2012. For the purpose of this evaluation, we have defined the intervention period (the exposure variable) based on the month and year that the woman delivered into three periods i.e. pre-intervention period (February 2011 to July 2012), early intervention period (August 2012 to December 2013) and late intervention period (January 2014 to March 2015).

### Outcome variables

We based this evaluation on four outcomes: 1) Attendance of at least four visits of ANC provided by a health professional or a health extension worker; 2) receipt of all three basic services during antenatal care: blood pressure measurement, blood sample taken, urine sample taken; 3) delivery assisted by a skilled birth attendant (SBA) i.e. a doctor, a nurse, a midwife, or a health officer; and 4) receipt of PNC within seven days of delivery by a health professional or a health extension worker.

### Other variables

The surveys collected data on district, urban/rural residence, woman’s age; parity; education level; marital status; ethnicity; and religion, index child’s age in months, partner’s education, and distance to the nearest health facility with maternity services. Data were also collected on attitude towards maternal health care, perceived quality of maternal health care at nearest health facility, knowledge of pregnancy danger signs, and birth preparedness. These later four variables were considered to be intermediate outcomes. We derived wealth index through factor analysis of household assets, housing material, and access to water and sanitation services. We used the first of the factor scores to represent the wealth index [20]. We derived maternal health attitude score using factor analysis of eight Likert scale questions that explored perceptions of women towards birth preparedness; male involvement in maternal health; and barriers to institutional childbirth as described elsewhere [12, 18].

### Statistical analysis

We analysed data in Stata version 12 using survey commands to account for the complex sampling design. We assessed the sociodemographic characteristics of women across the intervention periods using descriptive statistics and design based F tests. We cross tabulated the intermediate outcome variables namely: knowledge of pregnancy danger signs, attitude towards maternal health, perceived quality of care, attendance of any ANC and birth preparedness against the intervention periods and assessed linear trends across the periods.

To assess the effect of the intervention on each outcome variable, we used logistic regression models to obtain odds ratios (ORs) and 95 % confidence intervals (CIs). The ORs were adjusted for woman’s age, place of residence, wealth index tertile, parity, partner’s education, woman’s education and religion. We used the pre-intervention period as the reference category in all analyses. We explored for linear effects by entering, in the models, the intervention period as a continuous variable.

## Results

### Characteristics of women

A total of 999 women were surveyed. Women who delivered before and during the intervention periods were similar in terms of their sociodemographic characteristics as shown in Table 1. Table 2 shows the distribution of participants in the pre- and during intervention periods according to intermediate outcomes. The percentage of women who could mention at least three danger signs of pregnancy increased from 21.6 % in the pre-intervention period to 38.6 % in the late intervention period but overall, there was no significant association between the intervention and the number of danger signs mentioned. The proportion of women with better perception about the quality of maternal health services and with higher maternal health attitude score significantly increased during the intervention period (each, *P* < 0.001). There was also a significant increase in the proportion of women taking specific actions to prepare for the birth of the baby.

**Table 1:** Characteristics of women in the study sample by period of delivery before and after the start of the intervention

Characteristics	Period			*P* value*
	Pre-intervention (Feb 2011–Jul 2012)	Early intervention (Aug 2012–Dec 2013)	Late intervention (Jan 2014–Mar 2015)	
	(%) (*n* = 334)	(%) (*n* = 327)	(%) (*n* = 338)	
District				0.960
Wolisso	56.4	56.4	54.0	
Goro	14.0	16.4	17.2	
Wonchi	29.6	27.2	28.8	
Residence				0.521
Urban	12.8	16.6	19.8	
Rural	87.2	83.4	80.2	
Ethnicity				0.449
Oromo	88.1	85.3	81.9	
Other	11.9	14.7	18.1	
Wealth index tertile				0.328
Low	39.4	31.8	29.0	
Middle	29.9	37.0	33.2	
High	30.7	31.2	37.8	
Age in years				0.659
15–24	24.3	27.4	28.0	
25–29	37.0	36.3	33.5	
30–34	20.0	18.2	23.0	
35–49	18.7	18.1	15.5	
Parity				0.185
1	18.2	21.4	22.4	
2–3	29.0	30.9	25.2	
4–5	32.8	26.0	26.4	
> 5	20.0	21.7	26.0	
Woman’s education level				0.368
None	54.0	50.2	51.6	
Primary 1–4	21.8	16.5	16.9	
Primary 5–8	15.5	19.6	20.8	
Secondary or higher	8.7	13.8	10.7	
Partner’s education level				0.459
None/no partner	24.9	20.7	20.4	
Primary 1–4	22.5	23.5	19.1	
Primary 5–8	35.0	33.3	35.7	
Secondary or higher	17.6	22.4	24.8	
Marital status				0.546
Married	95.2	97.2	96.2	
Single	4.8	2.8	3.8	
Religion				0.462
Orthodox Christian	55.8	47.1	42.9	
Protestant	22.5	27.2	31.5	
Muslim	21.7	25.7	25.6	
Time to nearest facility				0.516
< 30 min	36.1	40.2	47.5	
30–59 min	24.8	21.2	18.7	
≥ 60 min	39.1	38.7	33.8	

*F test accounting for complex sampling design

**Table 2:** Intermediate maternal health outcomes by period of delivery before and after the start of the intervention

Intermediate outcomes	Period			*P* value for trend
	Pre-intervention (Feb 2011–Jul 2012)	Early intervention (Aug 2012–Dec 2013)	Late intervention (Jan 2014–Mar 2015)	
	% (*n* = 334)	% (*n* = 327)	% (*n* = 338)	
Number of pregnancy danger signs mentioned				0.186
0	23.6	28.0	22.1	
1–2	54.8	45.1	39.3	
≥ 3	21.6	26.9	38.6	
Perceived quality of care at nearest facility				<0.001
Average/poor/don’t know	37.3	34.0	22.9	
Good	47.3	42.0	41.4	
Very good	15.4	24.0	35.7	
Maternal health attitude score tertiles				<0.001
Low	44.9	28.5	17.8	
Medium	34.0	34.1	40.7	
High	21.1	37.4	41.5	
Number of birth preparation actions*				0.003
0	49.1	37.8	33.7	
1	38.8	44.9	47.4	
≥ 2	12.1	17.3	18.9	

*The list included: identify transport, save money, identify blood donor, decide delivery place and identify skilled provider

### Changes in outcomes

**Fig. 2:**
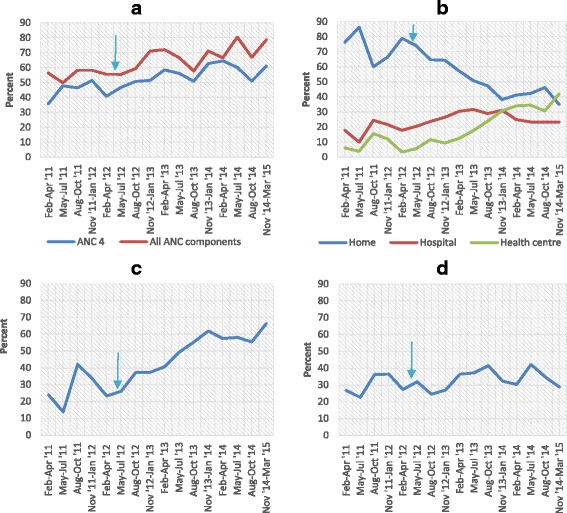
Trends in coverage of maternal health indicators. This figure shows the coverage of maternal health indictors by date of delivery. Part **a** shows coverage of at least four antenatal care visits and receipt of three ANC components; part **b** shows trends in place of delivery; part **c** shows coverage of skilled birth attendance; and part **d** shows coverage of postnatal care. The arrows indicate the start of the intervention period. On the x-axis, Feb–Apr’ 11 refers to February 2011 to April 2011, and so forth

Figure 2 shows trends in the coverage of at least four ANC visits and receipt of all three basic components of ANC (**a**), place of delivery (**b**) delivery by SBA (**c**) and PNC attendance (**d**). Overall, the figure suggests that over time, there was an increase in coverage of four ANC visits; receipt of ANC components; and delivery by SBA, but no change in PNC coverage. The greatest increase was in the coverage of delivery by SBA. The figure (part **b**) also shows that increased coverage of delivery by SBA was driven by increased delivery in HCs and not at the hospital where the proportion of deliveries remained virtually constant. The proportion of deliveries at HCs rose from 7.3 % in the pre-intervention period to 35.6 % in the late intervention period (*p* < 0.001, data not shown).

Results in Table 3 show that after adjusting for socio-demographic factors, there was a linear increase in the coverage of receipt of all three ANC components and delivery by a SBA from the pre-intervention period to the late intervention period. The odds of receiving all three components of ANC were twice higher in the late intervention period than in the pre-intervention period (OR 2.09; 95 % CI 1.12-3.89). Women in the late intervention period had a five-fold increase in the odds of SBA at delivery than those who delivered during the pre-intervention period (OR 5.04; 95 % CI 2.53-10.06). After accounting for sociodemographic factors, there was no significant change in the coverage of at least four ANC visits and PNC.

**Table 3:** Logistic regression analysis of changes in outcome indicators before (Feb 2011–Jul 2012) and after the start of the intervention

Indicator	Coverage % (*N* = 999)	Unadjusted OR (95 % CI)	*P* value for trend	Adjusted OR** (95 % CI)	*P* value for trend
Attended four ANC visits			0.011		0.100
Feb 2011–Jul 2012	44.8	1		1	
Aug 2012–Dec 2013	54.3	1.46 (1.01-2.10)		1.04 (0.67-1.60)	
Jan 2014–Mar 2015	59.2	1.78 (1.14-2.77)		1.83 (0.89-3.77)	
Received all three ANC components*			0.021		0.022
Feb 2011–Jul 2012	55.5	1		1	
Aug 2012–Dec 2013	66.1	1.56 (0.97-2.51)		1.42 (0.91-2.22)	
Jan 2014–Mar 2015	73.6	2.23 (1.13-4.43)		2.09 (1.12-3.89)	
Delivered by skilled birth attendant			<0.001		<0.001
Feb 2011–Jul 2012	26.5	1		1	
Aug 2012–Dec 2013	43.7	2.16 (1.30-3.58)		2.34 (1.42-3.86)	
Jan 2014–Mar 2015	60.4	4.24 (2.00-9.00)		5.04 (2.53-10.06)	
Received postnatal care			0.463		0.934
Feb 2011–Jul 2012	30.1	1		1	
Aug 2012–Dec 2013	30.8	1.04 (0.65-1.68)		0.90 (0.58-1.42)	
Jan 2014–Mar 2015	34.4	1.22 (0.70-2.15)		1.02 (0.60-1.73)	

*Blood pressure checked, urine sample taken, blood sample taken

**Adjusted for woman’s age, place of residence, wealth index tertile, parity, partner’s education, woman’s education and religion

## Discussion

This study evaluated a multifaceted maternal and child health project implemented by a non-governmental organisation. The results suggest that the project was associated with increased coverage of receipt of all three basic components of ANC and SBA at delivery, but not with four ANC visits and PNC. The effect on SBA at delivery and receipt of three basic components of ANC was probably partly mediated through better perception towards the quality of maternity care provided in health facilities, improved attitude towards maternal health and improved birth preparedness (for SBA at delivery). Coverage of SBA at delivery was driven by increased utilisation of HCs which before the intervention were largely underutilised; attending to only 7 % of total deliveries despite their large number. Receipt of three basic components of ANC can be taken as a proxy measurement of the quality of ANC, thus the improvement in this indicator suggests that the project improved access to quality ANC.

The project was biased towards strengthening HCs to provide maternal and child health care services. This is because the hospital was already being well utilised. A recent study has shown that although there is inequity in access to maternal health services in the intervention area, women utilising HCs were more likely to be poorer (and rural residents) than those utilising the zonal hospital [21]. For most women, HCs were the nearest facilities for accessing childbirth services, however, before the start of the project, these facilities were being grossly underutilised partly because of being perceived to be providing poor quality of care [12]. Over the course of the project, the percent of women who perceived that the quality of care at the nearest health facility was good more than doubled to 35 %. Thus, the results of this evaluation suggest that strengthening HCs to provide delivery services could be one of the effective ways of scaling up coverage of SBA at delivery; in line with the principles of primary health care [22].

The WHO recommends that pregnant women should attend a minimum of four ANC visits to allow for appropriate delivery of a complete package of ANC services [23]. Although the coverage of four ANC visits increased from 44.8 % before the project to 59.2 % later in the project, this increase was not significant after accounting for socio-demographic factors. This observation was unexpected given that it is easier to improve the coverage of services such as ANC and PNC that can be offered through outreaches than those, such as childbirth care, that are offered only in fixed health facilities [24]. Given that a steeper increase in ANC coverage seems to have occurred in the later months of the project, it could be that in the initial stages, more time and effort was spent on increasing childbirth in HCs through improving infrastructure, providing equipment and training health staff than on providing preventive services at HPs and in the community.

There is a big challenge in providing PNC in Ethiopia. In the 2011 DHS, only 50 % of women who delivered in health facilities received PNC within the first two days [5]. It is unclear why the coverage of PNC attendance did not change in the course of the project. In the study districts, women with uncomplicated deliveries are usually discharged after six hours, and they may not return within a few days to the health facility due to barriers, such as distance, that affect access to health services. Different strategies for PNC with varying challenges to mothers and providers exist [25]. To improve PNC coverage, in 2012, the Ethiopian Government adopted a mixture of facility-based and community-based PNC strategy, leveraging the efforts of HEWs. HEWs are required to visit the mother and the baby within 48 h after birth [26]. Despite this, a survey conducted in 2014 showed that only 0.8 % of women in Oromia received PNC within two days by a HEW [4]. In our study, only 1.4 % of the 999 women surveyed received PNC within seven days by a HEW. Thus, there is still a huge potential to increase PNC coverage through HEWs. Lack of improvement in PNC coverage could also be because the project may have placed more emphasis on intrapartum care, including provision of emergency obstetrics care at HCs, than on PNC. A community based intervention project that involved family meetings and labour and birth notification to a HEW led to 3- to 10-fold increases in PNC coverage over a 2-year period in rural Ethiopia [27]. Such an approach could be piloted in the study districts.

Some researchers have used coverage of SBA at delivery to classify health systems in Africa as follows: low health system context where SBA at delivery is less than 30 %; middle health system context where SBA at delivery is 30–60 %; and high health system context where SBA at delivery is >60 % [24]. Based on this classification, it can be argued that the project strengthened the health system to provide maternal and neonatal health services, but a lot still needs to be done to ensure continued progress and sustainability. Sustainability of donor funded projects is always of concern to the government, donors, implementing agencies and project beneficiaries. In this study, the sustainability of removal of user fees at hospital, which is private-not-for-profit, may be of concern. Nonetheless, even before the present project, user fees at the hospital were highly subsidized because the hospital was getting financial support from the government under a public-private partnership (PPP) framework [13]. CUAMM and the hospital will have to continue to negotiate with the Ministry of Health to ensure continuity of the PPP and its expansion to cover all maternal neonatal and child health services.

The project started off with higher baseline coverage indicators than the national averages probably because CUAMM had been supporting health service delivery in the districts for a while. For instance, the NGO had been supporting the running of the zonal referral hospital since the year 2001 [28]. In addition, for PNC, the higher coverage could be because we defined this indicator broadly (i.e. care within seven days of birth). The high coverage of SBA at delivery but low coverage of PNC implies gaps and/or missed opportunities in the continuum of care [29]. The findings highlight the need to investigate further why women deliver assisted by SBA but don’t receive PNC. The aim should be to mitigate missed opportunities and improve access to health care because most neonatal deaths occur during the postnatal period [30], and failure to provide high quality PNC to neonates can hamper efforts to reduce neonatal mortality.

This study has some limitations. The use of a historical control group did not allow us to adjust for secular trends in service use. Although we adjusted for socio-demographic factors that might affect health service utilisation independent of any intervention, Ethiopia has been experiencing a general nationwide increase in the coverage of maternal health services. For instance, at national level SBA at delivery increased from 10 % in 2011 to 16 % in 2014 [3, 4]. In our study, coverage of SBA at delivery more than doubled in a shorter time despite starting off at a higher baseline, which suggests that the project played a role in this. This study focused on coverage, yet coverage alone cannot reduce maternal and neonatal deaths if the quality of care provided is poor and women in the poorer strata are excluded. A study conducted at the hospital found that the quality of maternal health care at this facility was good [21], but little is known about the quality of care at HCs especially with regard to intrapartum and postpartum care. We defined PNC as care within seven days after childbirth; this is different from the international definition of PNC as care within two days. Data on PNC within two days were inadvertently not collected in the second survey. Thus, this indicator should be interpreted cautiously especially when making comparisons. Future evaluation should focus on a detailed evaluation of the quality of care at HCs given their increasing importance in service delivery as it emerged in this study. Because some of the questions asked during the interviews required women to recall events that occurred up to the past two years, our estimates may have been affected by recall bias. Finally, because this was a multifaceted project, we cannot associate any outcome with any particular component of the intervention.

## Conclusions

The maternal and child health project implemented by the NGO Doctors with Africa CUAMM in Wolisso, Goro and Wonchi districts in Ethiopia was associated with increased coverage of receipt of three basic components of ANC and delivery assisted by a SBA. There was however no increase in the coverage of at least four ANC visits or PNC after accounting for potentially confounding factors. Increase in coverage of SBA at delivery was driven by increased utilisation of childbirth services at HCs. More efforts, involving HEWs, are needed to increase the coverage of ANC and PNC.

## Additional file

Additional file 1: Table S1. Power calculations for comparison between outcomes in the pre-intervention period and the post intervention period. (DOCX 14 kb)

## Abbreviations

ANC: Antenatal care
CI: Confidence interval
CUAMM: Collegio Universitario Aspiranti Medici Missionari
DHS: Demographic and Health Survey
HC: Health Centre
HEW: Health Extension Worker
HP: Health Post
JHPIEGO: Johns Hopkins Program for International Education in Gynaecology and Obstetrics
MDG: Millenium Development Goal
MMR: Maternal mortality ratio
NGO: Non-governmental organisation
PNC: Postnatal care
OR: Odds ratio
SBA: Skilled birth attendant
UNICEF: United Nations Children’s Fund
