# Supplementary material for: Evaluation of a maternal health care project in South West Shoa Zone, Ethiopia: before-and-after comparison
Source: Reprod Health. 2016 Aug 20;13:95. doi: 10.1186/s12978-016-0213-1 (PMC4992297; doi:10.1186/s12978-016-0213-1)
Supplement: Additional file 1: Table S1. — Power calculations for comparison between outcomes in the pre-intervention period and the post intervention period. (DOCX 14 kb) [file 12978_2016_213_MOESM1_ESM.docx]

**Additional file 1: Table S1 Power calculations for comparison between outcomes in the pre-intervention period and the post intervention period**

| **Variable** | **Coverage in pre-intervention**  **period (N=334)** | **Coverage in late post intervention period**  **(N=338)** | **Alpha** | **Power** |
| --- | --- | --- | --- | --- |
| Attended four ANC visits | 44.8% | 59.2% | 0.05 | 95.7% |
| Received all three ANC components* | 55.5% | 73.6% | 0.05 | 99.8% |
| Delivered by skilled birth attendant | 26.5 | 60.4 | 0.05 | 100% |
| Received postnatal care | 30.1 | 34.4 | 0.05 | 19.7% |

*blood pressure checked, urine sample taken, blood sample taken
